# Computational Study Regarding Co_x_Fe_3−x_O_4_ Ferrite Nanoparticles with Tunable Magnetic Properties in Superparamagnetic Hyperthermia for Effective Alternative Cancer Therapy

**DOI:** 10.3390/nano11123294

**Published:** 2021-12-04

**Authors:** Costica Caizer

**Affiliations:** Department of Physics, West University of Timisoara, Bv. V. Pârvan No. 4, 300223 Timisoara, Romania; costica.caizer@e-uvt.ro

**Keywords:** Co-Fe ferrite nanoparticles, magnetic hyperthermia, specific loss power, optimization, alternative therapy, cancer

## Abstract

The efficacy in superparamagnetic hyperthermia (SPMHT) and its effectiveness in destroying tumors without affecting healthy tissues depend very much on the nanoparticles used. Considering the results previously obtained in SPMHT using magnetite and cobalt ferrite nanoparticles, in this paper we extend our study on Co_x_Fe_3−x_O_4_ nanoparticles for x = 0–1 in order to be used in SPMHT due to the multiple benefits in alternative cancer therapy. Due to the possibility of tuning the basic observables/parameters in SPMHT in a wide range of values by changing the concentration of Co^2+^ ions in the range 0–1, the issue explored by us is a very good strategy for increasing the efficiency and effectiveness of magnetic hyperthermia of tumors and reducing the toxicity levels. In this paper we studied by computational simulation the influence of Co^2+^ ion concentration in a very wide range of values (x = 0–1) on the specific loss power (*Ps*) in SPMHT and the nanoparticle diameter (*D_M_*) which leads to the maximum specific loss power (*P_sM_*). We also determined the maximum specific loss power for the allowable biological limit (*P_sM_*)*_l_* which doesn’t affect healthy tissues, and how it influences the change in the concentration of Co^2+^ ions. Based on the results obtained, we established the values for concentrations (x), nanoparticle diameter (*D_M_*), amplitude (*H*) and frequency (*f*) of the magnetic field for which SPMHT with Co_x_Fe_3−x_O_4_ nanoparticles can be applied under optimal conditions within the allowable biological range. The obtained results allow the obtaining a maximum efficacy in alternative and non-invasive tumor therapy for the practical implementation of SPMHT with Co_x_Fe_3−x_O_4_ nanoparticles.

## 1. Introduction

The magnetic nanoparticles most often used in magnetic hyperthermia therapy in the ferrimagnetic materials class are those of iron oxide due to their good magnetic characteristics and their efficient use at high frequencies. Of these materials, Fe_3_O_4_ nanoparticles (magnetite) are still the most used [1,2,3,4,5,6,7,8,9,10,11,12,13,14,15,16,17] due to their great magnetic properties for magnetic hyperthermia [18,19,20], and also their low toxicity towards cells [21].

However, extensive studies have been conducted on the subject [22,23,24,25,26,27,28,29,30] with the aim of finding other magnetic nanoparticles and magnetic nanomaterials/nanostructures suitable for use in magnetic hyperthermia, with improved properties. In this regard, of particular interest are cobalt ferrite and cobalt ferrite nanoparticles, Co_x_Fe_3−x_O_4_, in order to be applied in magnetic or superparamagnetic hyperthermia due to their magnetic anisotropy which is very different from that of magnetite [18,19], and could lead to substantial improvements in terms of magnetic or superparamagnetic hyperthermia. In terms of magnetic anisotropy, CoFe_2_O_4_ ferrite is magnetically hard, having a magnetocrystalline anisotropy constant of 200 × 10^3^ J/m^3^, while Fe_3_O_4_ ferrite (magnetite) is magnetically soft, having an anisotropy constant of only 11 × 10^3^ J/m^3^, although their spontaneous magnetizations (*M_s_*) differ only slightly from each other (*M_s_* = 480 kA/m for Fe_3_O_4_ and *M_s_* = 425 kA/m for CoFe_2_O_4_) [18].

In superparamagnetic hyperthermia (SPMHT), the very high magnetic anisotropy of CoFe_3_O_4_ ferrite nanoparticles compared to that of F_3_O_4_ magnetite, radically influences the hyperthermia effect, which is reflected in the specific loss power and, finally, on the heating temperature of the nanoparticles [31,32]. As a result, the maximum effect in SPMHT given by the specific loss power is obtained in the case of soft nanoparticles of Fe_3_O_4_ for a diameter (size) of nanoparticles (approximate spherical) of ~16 nm, and in the case of CoFe_2_O_4_ hard ferrite nanoparticles for a diameter of only ~6 nm (the exact value depending on the frequency of the alternating magnetic field). These nanoparticle sizes, in terms of SPMHT which uses superparamagnetic nanoparticles would be too large for Fe_3_O_4_ nanoparticles and too small for CoFe_2_O_4_ nanoparticles, both types of nanoparticles thus having advantages and disadvantages in magnetic hyperthermia for cancer therapy. More detailed results and discussions on these issues were previously presented [31,32].

Considering the above and the sporadic results of the overall research on the matter so far, lacking a systematic approach, we’ve focused on studying SPMHT on Co_x_Fe_3−x_O_4_ nanoparticles for the entire range of values x = 0–1, wherein the bivalent Fe^2+^ ions are replaced by a percentage of Co^2+^ ions (x) in the octahedral lattice of the spinel of Fe^3+^[Co_x_^2+^Fe_(1−x)_^2+^, Fe^3+^]O_4_^2-^ ferite (the right bracket comprises the Fe^3+^, Fe^2+^ ions and Co^2+^ from the octahedral lattice, and outside the parentheses are the Fe^3+^ ions from the tetrahedral lattice within the ferrite structure [18]). Thus, by replacing Fe^2+^ ions with Co^2+^ ions in the entire range of atomic percentage values (0–1), starting from Fe_3_O_4_ magnetite (for x = 0) and reaching the CoF_2_O_4_ ferrite for x = 1, the magnetic anisotropy will change in a very wide range of values, and thus different Co_x_Fe_3−x_O_4_ nanoparticles which have different magnetic characteristics in magnetic hyperthermia depending on the concentration of Co^2+^ ions can be obtained. Therefore, we can modify the parameter x (concentration of Co^2+^ ions in the structure of magnetite) in order to obtain adjustable properties in superparamagnetic hyperthermia, thus, being able to find the optimal values of the parameters which give the best results in magnetic hyperthermia. With this in mind, we focused on a systematic study by using a 3D/2D computational tools and as complete as possible in terms of the specific loss power in magnetic hyperthermia (which is the key value that indicates whether the nanoparticles are good or not to obtain the maximum hyperthermia effect) depending on the concentration of Co^2+^ ions (x), in order to find which nanoparticles would give the best results in SPMHT. At the same time, we studied the maximum specific loss power for the admissible biological limit (without affecting healthy tissues), depending on the concentration of Co^2+^ (x) ions, in order to optimize SPMHT with Co_x_Fe_3−x_O_4_ for its practical implementation in vivo and, in the future, in clinical trials with maximum efficacy.

## 2. Theoretical Considerations on Specific Loss Power in Superparamagnetic Hyperthermia

In superparamagnetic hyperthermia (SPMHT) of tumors with magnetic nanoparticles [14,17,31,33] the basic mechanism that leads to the heating of dispersed and fixed nanoparticles in the tumor are the Néel magnetic relaxation processes [8,9,34]. Thus, under the action of an alternating magnetic field with a frequency in the range of hundreds of kHz [20], superparamagnetic (biocompatible) nanoparticles dispersed in the tumor by different techniques, heat to temperatures of 42–43 °C, thus, leading to the irreversible destruction of tumor cells by apoptosis [35]. However, the efficiency of the method depends very much on the type of magnetic nanoparticles used for this therapy and the magnetic relaxation processes that take place in the alternating magnetic field.

The specific loss power (*P_s_*) in magnetic nanoparticles in the presence of an alternating magnetic field with frequency *f* and amplitude *H* is [20,36]:

(1) $$P_{s}=\frac{\pi \mu_{0}\chi^{\prime \prime }}{\rho }fH^{2}\;$$

where *ρ* is the density of the magnetic material, $\mu_{0}$ is the magnetic permeability of the vacuum and $\chi^{\prime \prime }$ is the imaginary component of complex magnetic susceptibility, given by the following expression:

(2) $$\chi =\chi^{\prime }-j\chi^{\prime \prime }\;$$

According to Debye’s theory, the components of complex magnetic susceptibility are given by the relations [20,37,38]

(3) $$\chi^{\prime }=\chi_{0}\frac{1}{1+{\left(\omega \tau \right)}^{2}}\;$$

and:

(4) $$\chi^{\prime \prime }=\chi_{0}\frac{\omega \tau }{1+{\left(\omega \tau \right)}^{2}}\;$$

where $\chi_{0}$ is the static magnetic susceptibility, *τ* is the magnetic relaxation time, and *ω* is the pulsation of the alternating magnetic field (ω = 2πf).

Static magnetic susceptibility in the case of magnetization of superparamagnetic nanoparticles [39], according to Langevin’s law of magnetization:

(5) $$M=M_{sat}\left(\coth\xi -\frac{1}{\xi }\right)\;$$

is given by:

(6) $$\chi_{0}=\frac{3\chi_{i}}{\xi }\left(\coth\xi -\frac{1}{\xi }\right)\;$$

where $\chi_{i}$ is the initial magnetic susceptibility:

(7) $$\chi_{i}=\frac{\varepsilon \pi \mu_{0}M_{s}^{2}D^{3}}{18k_{B}T}\;$$

and the parenthesis from Equation (6) is the Langevin function in the case of magnetic nanoparticles [39,40] having the argument:

(8) $$\xi =\frac{\pi \mu_{0}M_{s}D^{3}}{6k_{B}T}H\;$$

In Equations (5), (7) and (8), *M_sat_* is the saturation magnetization, *D* is the diameter of the nanoparticles (approximate spherical), *M_s_* is the spontaneous magnetization, *ε* is the packing fraction of nanoparticles *k_B_* is Boltzmann’s constant, and *T* is the temperature. In the case of nanoparticle systems, the magnetic packing fraction expressed by the observable *ε* in Equation (7) must also be taken into account.

The magnetic relaxation time, according to Néel’s theory [34], is:

(9) $$\tau =\tau_{0}exp\left(\frac{\pi KD^{3}}{6k_{B}T}\right)\;$$

where *K* is the magnetic anisotropy constant, and *τ_0_* is a time constant which usually has a value of 10^−9^ s [41].

Thus, taking into account all the above formulas, the specific loss power in the magnetic nanoparticles in an alternating magnetic field (harmonic) with frequency *f* and amplitude *H*, will have the expression [31]:

(10) $$P_{s}=\frac{3\pi \mu_{0}\chi_{i}}{\rho \xi }\left(\coth\xi -\frac{1}{\xi }\right)\frac{2\pi f\tau }{1+{\left(2\pi f\tau \right)}^{2}}fH^{2}\;\left(W/g\right)\;$$

This equation and the above will be used in our 3D/2D computational study considering the specific loss power in Co_x_Fe_3−x_O_4_ nanoparticles for x = 0–1, as a function of the characteristic observables of the magnetic nanoparticles and the parameters of the alternating magnetic field. The key parameters considered in our study are the size (diameter) of the nanoparticles (*D*), the concentration of Co^2+^ (x) ions (which determines a certain magnetic anisotropy), and the alternating magnetic field parameters, amplitude (*H*) and frequency (*f*), on which will depend to a large extent the specific loss power (*P_s_*) and the efficiency of the SPMHT method. The ultimate goal of the study is to find the optimal observables/parameters that lead to a maximum specific loss power in the biological allowable limit (*P_sM_*)*_l_*, so that the SPMHT method can be applied with maximum effectiveness in tumor therapy in vitro, in vivo and then in the future in clinical trials.

## 3. Results and Discution

### 3.1. Characteristic Observables of Nanoparticles Depending on the Concentration of Co^2+^ Ions and Alternating Magnetic Field Parameters, and Input/Output Data Used in SPMHT

#### 3.1.1. Magnetic Anisotropy and Spontaneous Magnetization

**Figure 1:**
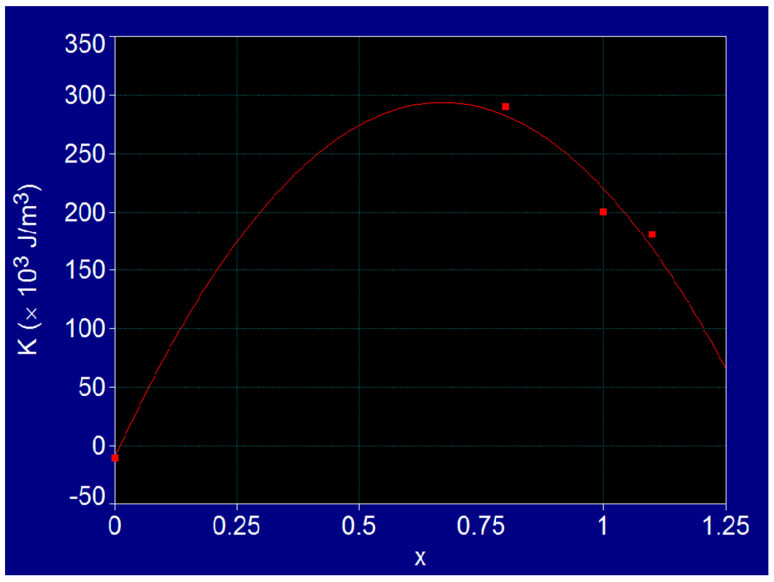
Fit curve on experimental reference values for magnetic anisotropy constant of Co_x_Fe_3−x_O_4_ ferrite.

**Figure 2:**
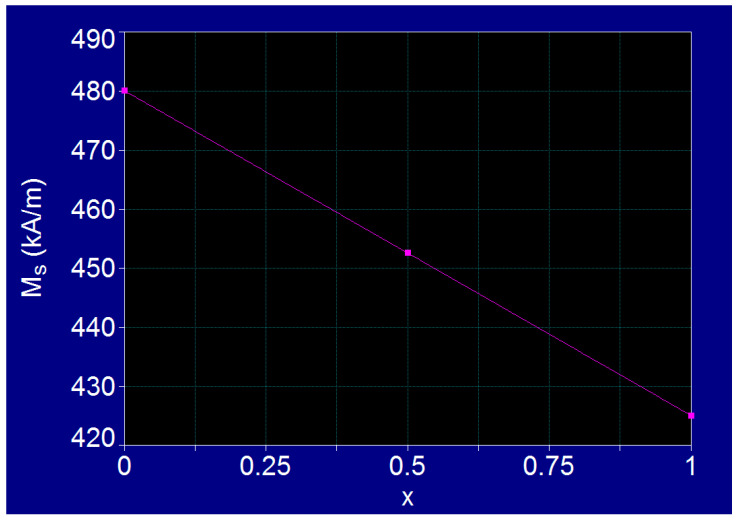
Fit on experimental reference values for spontaneous magnetisation of Co_x_Fe_3−x_O_4_ ferrite.

In the case of cobalt-iron ferrite (Co-Fe) with the chemical formula Co_x_Fe_3−x_O_4_ for x = 0–1, depending on the concentration x of Co^2+^ ions in the structure of Fe^3+^[Co_x_^2+^Fe_(3−x)_^2+^, Fe^3+^]O_4_^2−^ ferrite, where the bivalent Co^2+^ ions occupy the octahedral positions in the spinel structure, the values of the magnetic anisotropy constant and of the saturation magnetization at room temperature were determined by fitting the experimental reference data [18,19] (Figure 1 and Figure 2). These values extracted from the fit curves for different values of concentration x, are given in Table 1.

Thus, a very important result was found for the magnetocrystalline anisotropy constant *K* of the Co_x_Fe_3−x_O_4_ ferrite, which varies in very wide limits when the concentration of Co^2+^ ions changes from the value 0, corresponding to the spinel Fe_3_O_4_ (magnetite) with *K* = 11 × 10^3^ J/m^3^, to value 1, corresponding to the cobalt ferrite CoFe_2_O_4_ with *K* = 200 × 10^3^ J/m^3^. Also, it is observed that the variation of the anisotropy constant presents a maximum (294 × 10^3^ J/m^3^) at the value x = 0.67, and when the concentration x decreases to 0 the constant *K* decreases to the value corresponding to the magnetite (11 × 10^3^ J/m^3^) (Figure 1).

In order to determine the sponteneous magnetization of the Co_x_Fe_3−x_O_4_ ferrite as a function of the concentration of Co^2+^ ions for the variation of x in the range (0–1), we considered a linear variation of it with the concentration (x) of Co^2+^ ions (Figure 2). Thus, the values for spontaneous magnetization in Table 1 are obtained. However, in the case of nanoparticles the values may sometimes differ depending on the preparation method, nanoparticle size, type of material, etc. Therefore, in order not to cause confusion in our study for determining the values for *K* and *M_s_* by fitting we used the well known standard values for Co-Fe [18,19] ferrite. For a better accuracy in the applications of magnetic hyperthermia it is beneficial to determine experimentally the effective values of the magnetic anisotropy constant and the saturation magnetisation of nanoparticles that will be used.

#### 3.1.2. Nanoparticles and Alternating Magnetic Field Parameters

Having in view that the values of nanoparticle diameters (*D*) corresponding to the maximum loss power (*P_sM_)* in magnetic hyperthermia are ~16 nm for magnetite and ~6 nm for cobalt ferrite, we considered for this study the range of interest to be 1–20 nm (Table 2) for the nanoparticle size.

For our study on specific loss power, we considered the nanoparticles to be spherical and the value of the packing volumetric fraction (*ε*) in Table 2 (which are the most commonly used in magnetic and superparamagnetic hyperthermia). Also, based on our previous results [31,32], we also considered the amplitude (*H*) and frequency (*f*) of the alternating magnetic field in the ranges given in Table 2.

#### 3.1.3. Input and Output Data Used in Computational Study of SPMHT

For computational study using Co_x_Fe_3−x_O_4_ (x = 0–1) nanoparticles we used a professional software for 3D/2D calculus and representation. The input data are the characteristic observable of nanoparticles and the parameters of magnetic field from Table 2, and the magnetic observables of nanoparticles as a function of Co^2+^ ions concentration (x) from Table 1. The output data is mainly the specific loss power expressed by Equation (10) with Equations (1)–(9). The aim is to determine the specific loss power by Co_x_Fe_3−x_O_4_ nanoparticles for different concentrations of Co^2+^ ions depending on their size and alternating magnetic field parameters. At the same time, the maximum specific loss power for the admissible biological limit was determined.

### 3.2. The Specific Loss Power in Superparamagnetic Hyperthermia with Co_x_Fe_3−x_O_4_ Ferrite Nanoparticles

**Figure 3:**
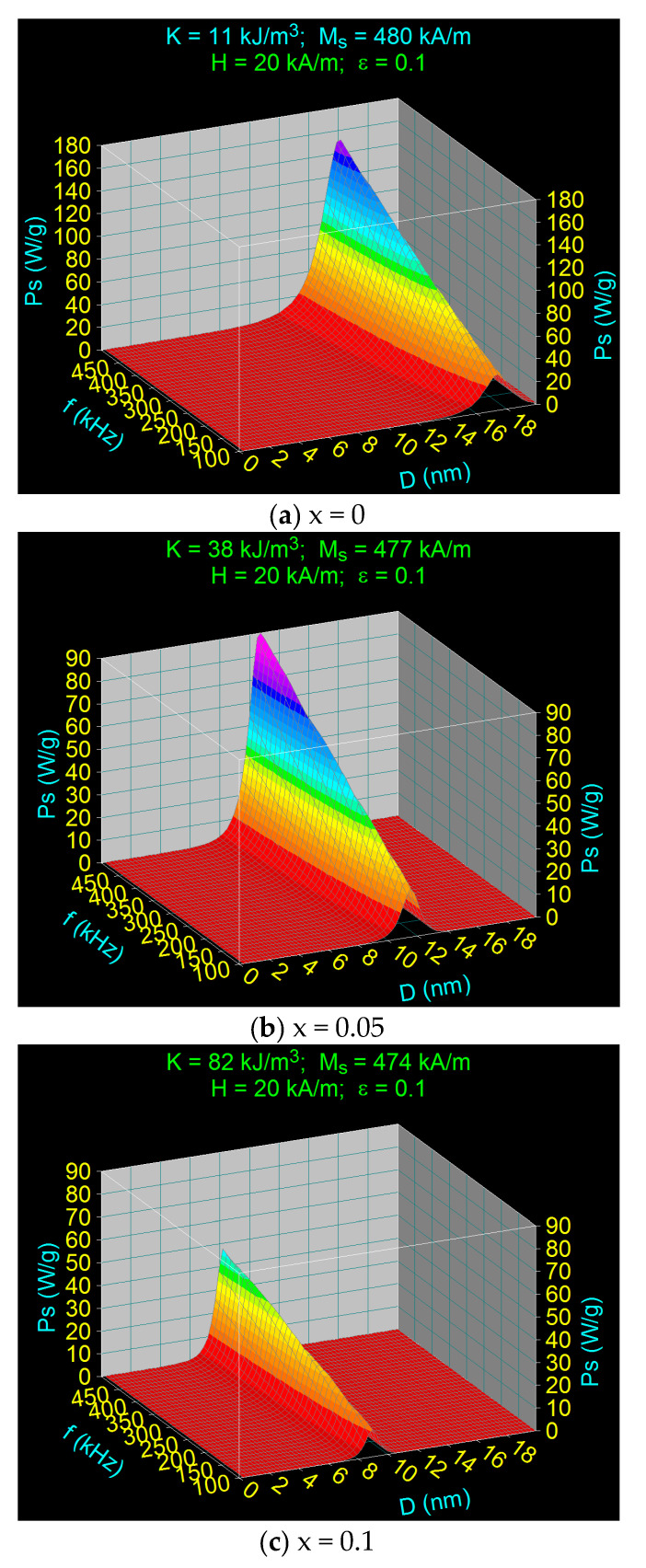

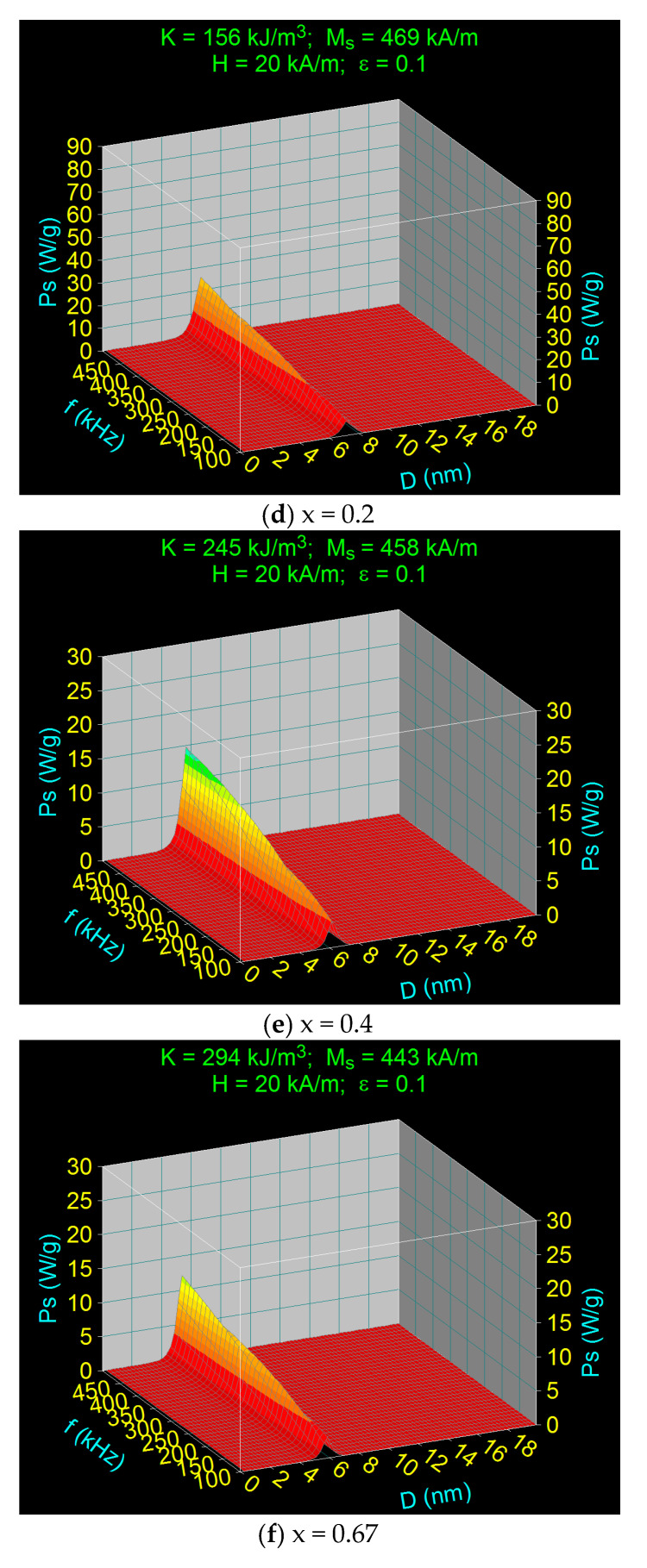

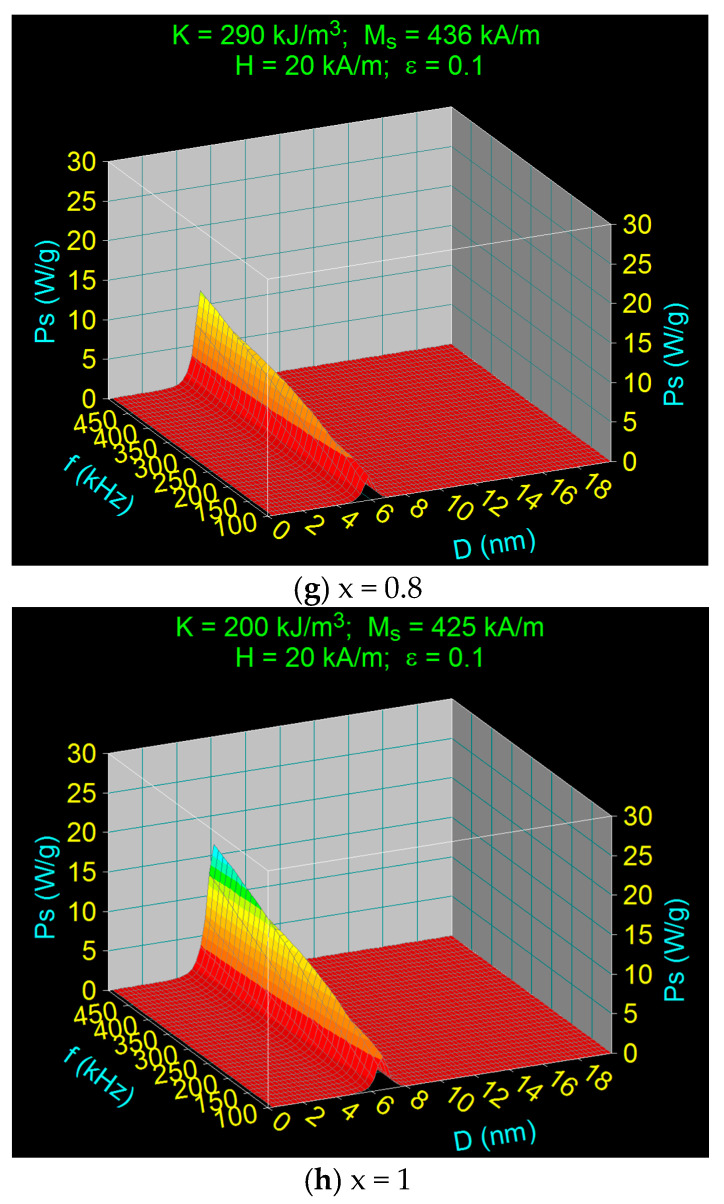
Specific loss power variation in the case of Co_x_Fe_3−x_O_4_ ferrimagnetic nanoparticles as a function of the diameter of nanoparticles and the magnetic field frequency for different Co^2+^ ions concentration: (**a**) 0, (**b**) 0.05, (**c**) 0.1 (**d**) 0.2, (**e**) 0.4), (**f**) 0.67, (**g**) 0.8, and (**h**) 1.

Using Equation (10) with the observables given by Equations (7)–(9), we calculated the specific loss power in the case of Co_x_Fe_3−x_O_4_ ferrimagnetic nanoparticles for the values of the concentration of Co^2+^ ions (x) located in the range of x = 0–1 (Table 1). The specific loss powers determined are 3D shown in Figure 3, as a function of nanoparticle diameter (*D*) and magnetic field frequency (*f*), for a constant magnetic field of 20 kA/m. For each value of the concentration x, the corresponding values for the magnetic anisotropy constant *K* and the saturation magnetization *M_s_* given in Table 1 were used.

**Figure 4:**
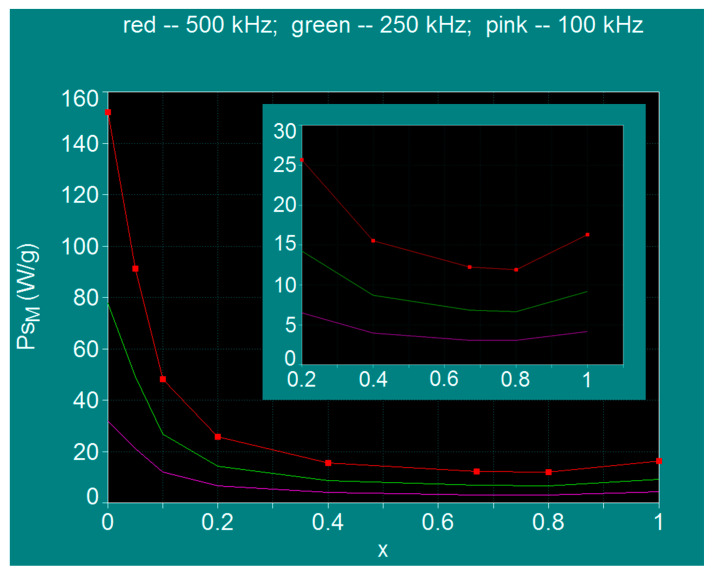
Maximum specific loss power variation in the case of Co_x_Fe_3−x_O_4_ ferrimagnetic nanoparticles as a function of the Co^2+^ ions concentration. Inset: magnified image for the range x = 0.2–1.

The results obtained and shown in diagrams (a)–(h) of Figure 3 are summarized in Table 3, Table 4 and Table 5. Figure 3 shows the presence of the maximum specific loss power at a certain values of the nanoparticle diameter (*D_M_*), which is a critical parameter; the maximum specific loss power *P_sM_* decrease rapidly to zero for values slightly larger or smaller than the *D_M_* diameter. Also, in all cases, the maximum specific loss power increases with the frequency of the magnetic field (100–500 kHz) as shown in diagrams (a)–(h). These variations of the specific loss power are in agreement with the variations previously observed in the case of nanoparticles of Fe_3_O_4_ and CoFe_2_O_4_ [31,32]. However, a special result obtained now is that the maximum specific loss power *P_sM_* decreases continuously and rapidly at the beginning with the increase of the concentration of Co^2+^ ions (x) from x = 0 to x = 0.8 after which a slow increase is obtained until x = 1 (Figure 4). Thus, the maximum specific loss power *P_sM_* has a minimum at the concentration value of x = 0.8.

**Figure 5:**
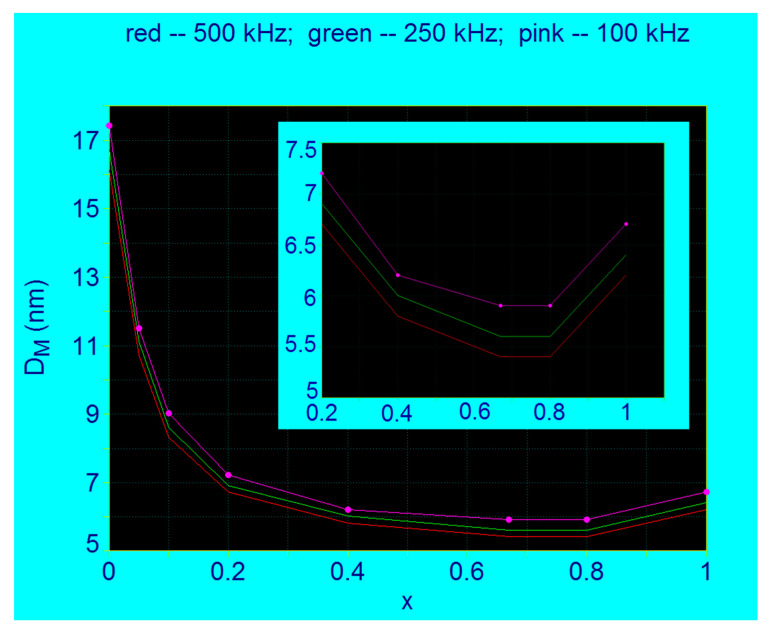
The variation of the nanoparticle diameters corresponding to the maximum specific loss power in the case of Co_x_Fe_3−x_O_4_ ferrimagnetic nanoparticles as a function of the Co^2+^ ions concentration. Inset: magnified image in the range x = 0.2–1.

A similar variation of the concentration x is also obtained for the diameters of nanoparticles *D_M_* corresponding to the maximum specific loss power *P_sM_* (Figure 5). The diameter of nanoparticles that give the maximum of the specific loss power decreases very much with the increase of the concentration x; e.g., for the frequency of 500 kHz the diameter of nanoparticles decreases from 16.1 nm for x = 0 to 6.2 nm when x increases to 1, the diameter also having a minimum value, which is 5.4 nm for x = 0.8.

This result, as well as the one regarding the specific loss power, are very important from the SPMHT point of view, because it suggests that it is possible to tune the observables and parameters of interest in magnetic hyperthermia, such as the specific loss power, nanoparticle size, and implicitly heating temperature and toxicity, magnetic packing fraction, etc., in a very wide range of values, just by simply changing the concentration of Co^2+^ ions. Thus, by changing the x concentration, the most suitable conditions can be obtained for the application of SPMHT in optimal conditions: obtaining maximum efficiency in SPMHT, obtaining maximum effectiveness in destroying tumor cells, obtaining minimum cellular toxicity on healthy tissues, or even lack of toxicity. This is a very important result for the practical implementation of SPMHT in vitro, in vivo and in future clinical trials.

By extracting the values of the maximum specific loss power *P_sM_* and those of the diameters of nanoparticles *D_M_* corresponding to the maximum powers, for each value of the concentration x in the range 0–1 (Table 1) at the frequencies of 100 kHz, 250 kHz and 500 kHz, the values from Table 3, Table 4 and Table 5 were obtained. Then, representing the powers *P_sM_* and the diameters *D_M_* as a function of the concentration x, the curves shown in Figure 4 and Figure 5 were obtained.

From the curves in Figure 4 it is observed that for all the frequencies considered (100, 250 and 300 kHz) the maximum specific loss power *P_sM_* in SPMHT decreases rapidly when the concentration of Co^2+^ ions increases in the range x = 0–0.2, and then the power decreases very slowly until x = 0.8. Then, when the concentration increases in the range x = 0.8–1 the power *P_sM_* also increases, but slowly. Thus, in the range of values x = 0.2–1 the variation of maximum loss power *P_sM_* presents a very wide minimum, having the lowest value at x = 0.8, depending on the value of frequency (inset of Figure 4). When the frequency decreases from 500 kHz to 100 kHz, the shape of the curves is preserved, only the power values decrease with the decrease of alternating magnetic field frequency (Figure 4, Table 3, Table 4 and Table 5).

Such a variation is also obtained for the diameter *D_M_* (Figure 5) which determines the maximum specific loss power *P_sM_*. From the point of view of magnetic hyperthermia both ranges of Co^2+^ ions concentration, both for x = 0–2 and for x = 0.2–1 are of interest and must be taken into account. If in the range x = 0–0.2 it must be borne in mind that the maximum specific loss power *P_sM_* decreases rapidly with increasing concentration of Co^2+^ ions, which could sometimes be a disadvantage in terms of power obtained in magnetic hyperthermia (which it will also be reflected on the heating temperature), in the next interval x = 0.2–1 the specific loss power *P_sM_* changes only slightly, which would be an advantage in magnetic hyperthermia. Thus, it results that it could be used in obtaining nanoparticles much different concentrations for Co^2+^ ions, in the range 0.2–1 respectively, without the maximum specific loss power to change too much in SPMHT. In addition, this could be another advantage in terms of the size of magnetic nanoparticles, sizes that are much smaller in this range (5.5–7 nm, depending on the frequency) (inset of Figure 5), and which lead to beneficial effect on reduction of cellular toxicity (due to the small size of the nanoparticles). Moreover, the much smaller size of the nanoparticles for the x = 0.2–1 range is also very beneficial in order to obtain intracellular hyperthermia, which is much more efficient in destroying tumor cells. The nanoparticles, being very small, penetrate much more easily into the cell (cytoplasm or even the nucleus) through the cell membrane, and thus destroying the tumor cells by magnetic hyperthermia much more efficiently inside them. At the same time, in this range of concentrations x = 0.2–1 the size of the nanoparticles does not change much (Figure 5), obtaining practically the same effect by magnetic hyperthermia for very different concentrations of Co^2+^ ions.

However, for the concentration range x = 0–0.2 the diameter of the nanoparticles changes a lot; e.g., at 500 kHz the *D_M_* diameter decreases from ~17 nm to ~7 nm when the x concentration increases from 0 to 0.2. This seems to be a disadvantage in terms of the power obtained in magnetic hyperthermia, and finally, the efficient heating and temperature obtained. However, if the power does not fall below a certain value, which is required in magnetic hyperthermia to heat the nanoparticles sufficiently, then the apparent disadvantage can be turned into a great advantage, namely: by changing the concentration of Co^2+^ ions in the range x = 0–0.2 the different diameters (sizes) of nanoparticles can be obtained in a wide range of values (7–17 nm) which will lead to different maximums of specific loss power in a very wide range of values. Thus, the best conditions can be found regarding the nanoparticle sizes, specific loss power, heating temperature, toxicity on healthy cells, etc., for SPMHT application in optimal conditions, by simply changing the concentration of Co^2+^ ions in the field of x = 0–0.2.

**Figure 6:**
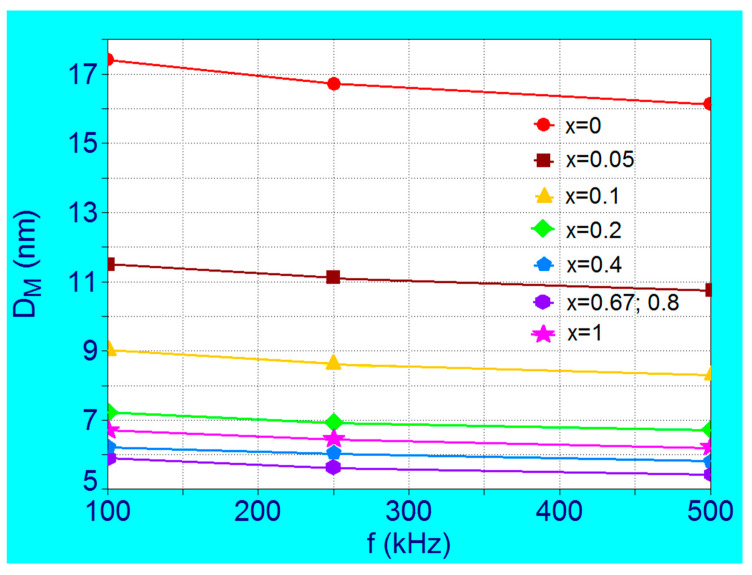
The variation of the nanoparticle diameters corresponding to the maximum specific loss power in the case of Co_x_Fe_3−x_O_4_ ferrimagnetic nanoparticles as a function of frequency for different Co^2+^ ions concentrations in the range x = 0–1.

Another important aspect that must take into consideration in the implementation of SPMHT is that the values of nanoparticle diameters *D_M_* that give the maximum specific loss power *P_sM_* depend on the frequency of alternating magnetic field besides the concentration of Co^2+^ ions, as shown in Figure 6.

When the frequency in SPMHT increases from 100 kHz to 500 KHz the diameter of nanoparticles *D_M_* that give the maximum specific loss power (*P_sM_*) decreases slightly for each value of the concentration x when it increases from 0 to 1. The decrease in diameter is more pronounced for the concentrations located in the range x = 0–0.2, being the largest at x = 0 where diameter *D_M_* decreases by 1.3 nm (from 17.4 nm to 16.1 nm) (Table 3 and Table 5). The *D_M_* values at each concentration must be taken into account for each frequency value used in the SPMHT in order to obtain the maximum loss power and the maximum thermal effect. Otherwise, the diameter being a critical parameter (Figure 3), a value slightly lower or slightly higher than that corresponding to *D_M_* value can greatly reduce the specific loss power, with negative effects on the hyperthermia effect, and consequently on the efficiency of SPMHT in tumor cells destruction.

### 3.3. Superparamagnetic Hyperthermia Optimization with Co_x_Fe_3−x_O_4_ Ferrite Nanoparticles: The Optimal Conditions Determination for Biologic Limit

Based on our previous results [31,32] we used the range of study for the magnetic field of 10–50 kA/m and the limit frequencies corresponding to these values, which results from the condition [42]:

(11) $$H\times f=5\times 10^{9}\;\mathrm{AHz}/m$$

**Figure 7:**
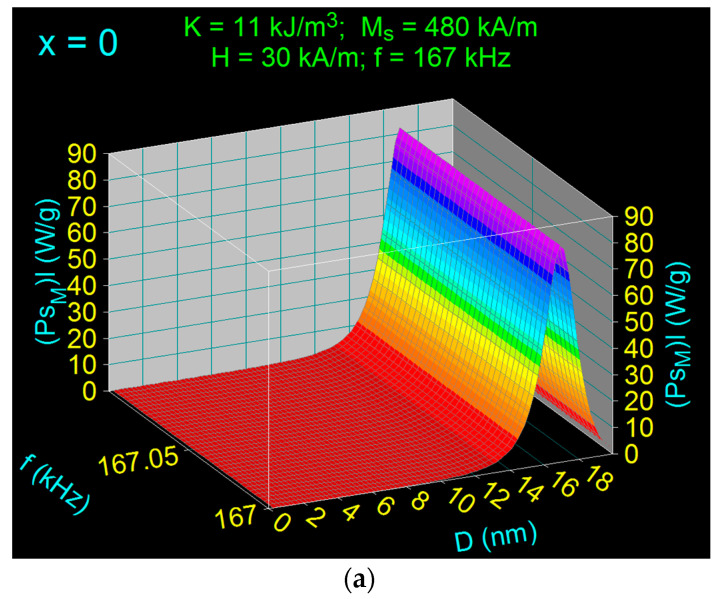

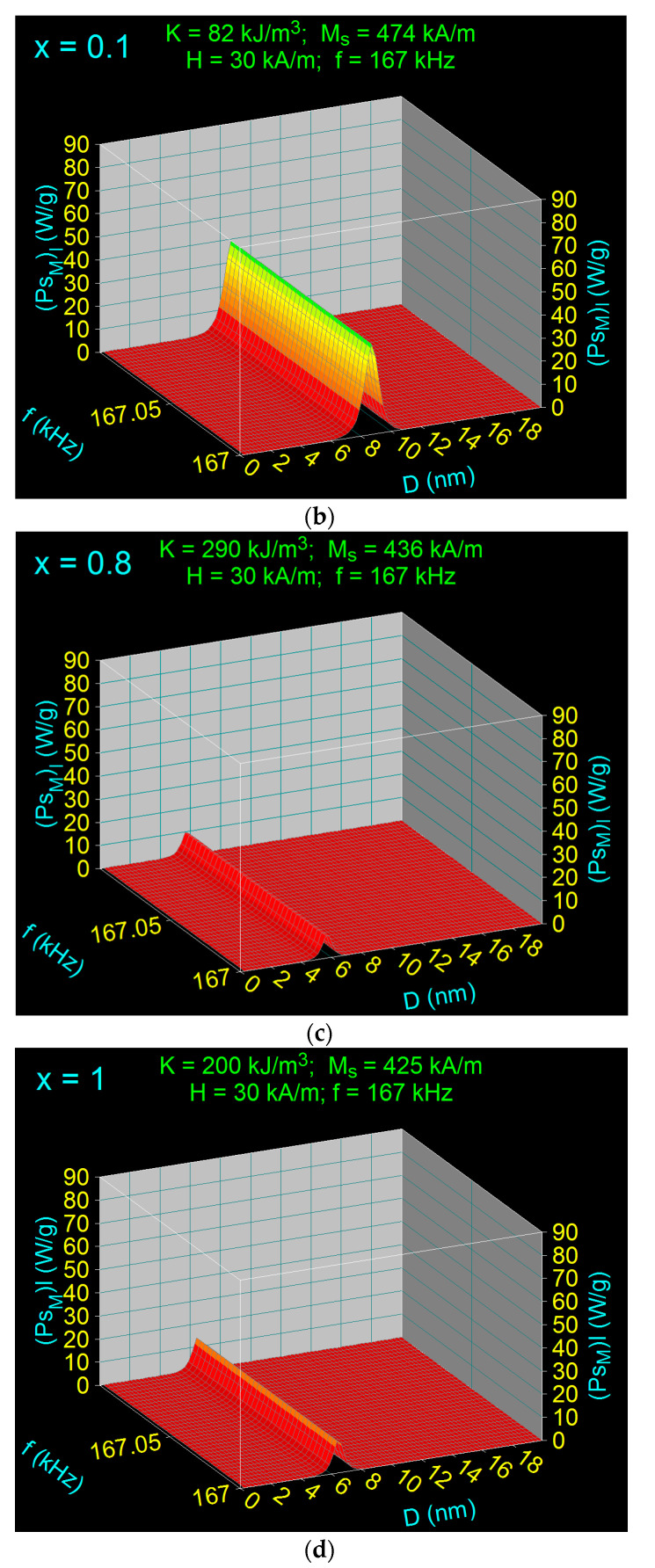
Specific loss power in the case of Co_x_Fe_3−x_O_4_ nanoparticles as a function of diameters for admissible biological limit (*H* = 30 kA/m and *f* = 167 kHz) and different Co^2+^ ions concentration (x): (**a**) 0, (**b**) 0.1, (**c**) 0.8, and (**d**) 1.

Using diagrams such as those in Figure 3 for all optimal magnetic fields (*H_o_*) and their corresponding frequencies for the allowable biological limit (*f_l_*), we determined the specific loss power for the allowable biological limit (*P_s_*)*_l_* depending on the concentration of Co^2+^ ions in the considered range (x = 0–1). The diagrams obtained for x = 0; 0.1; 0.8 and 1, and for a field of 30 kA/m, and a limit frequency of 167 kHz, are shown in Figure 7. The maximum values of the specific loss power for the admissible biological limit (*P_sM_*)*_l_* and those of the nanoparticle diameters (*D_Mo_*) that give the maximum loss power (*P_sM_*)*_l_* under the given conditions were extracted from diagrams like those in Figure 7, for *H_o_* = 10−50 kHz, *f_l_* = 100–500 kHz and x = 0–1. All values obtained for (*P_sM_*)*_1_* and *D_Mo_* are shown in Table 6. The very important result obtained is how the maximum specific loss power (*P_sM_*)*_l_* depends on the amplitude of the applied magnetic field for the admissible biological limit.

**Figure 8:**
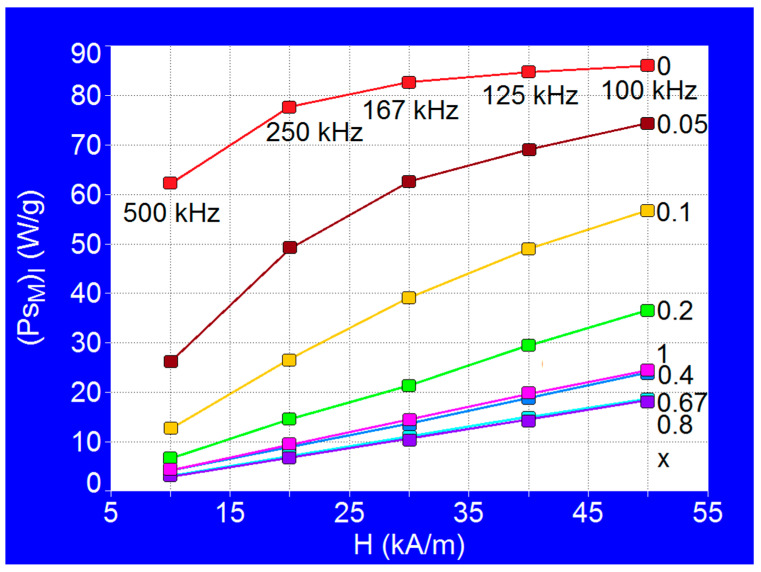
Maximum specific loss power for admissible biological limit (*P_sM_*)*_l_* in the case of Co_x_Fe_3−x_O_4_ (x = 0–1) nanoparticles as a function of the amplitudes of magnetic field, and for different limit frequencies.

Thus, the variations of power (*P_sM_*)*_l_* depending on the amplitude of magnetic field for different x concentrations of Co^2+^ ions are shown in Figure 8. The obtained results show a progressive decrease of the power (*P_sM_*)*_l_* with the increase of the concentration x of the Co^2+^ ions, decrease which is more accentuated for the first part of concentration range x = 0–0.2. Also, for x = 0.67 and x = 0.8 the power values (*P_sM_*)*_l_* are the lowest for the considered magnetic fields, being approximately the same for the two concentrations (0.67; 0.8) (Table 6), the powers (*P_sM_*)*_l_* increasing linearly with the magnetic field up to the value of 50 kA/m. Moreover, it is also observed that for the values x = 0.4 and x = 1 the approximately same powers (*P_sM_*)*_l_* are obtained, but slightly higher than in the previous case.

Considering our previous results [32] and considering for the efficient heating of the nanoparticles a value of the power (*P_sM_*)*_l_* higher of 10–15 W/g, we have established the optimal domains for magnetic field and concentrations which are recommended for the efficient practical implementation of SPMHT in this case. As seen in Figure 8, for x = 0 and magnetic fields greater than 25–30 kA/m the power (*P_sM_*)*_l_* increases only slightly, reaching a saturation level, so the use of larger magnetic fields is not justified (it does not lead to any significant increase in power). A similar effect is obtained in the case of x = 0.05 for magnetic fields greater than 40 kA/m. For x = 0.1 the power saturation effect occurs at fields greater than 50–60 kA/m. For values of concentration x greater than 0.2 and up to 1 the saturation is obtained at much higher values of the magnetic field, and up to 50 kA/m the variations of the power (*P_sM_*)*_l_* with concentration x are approximately linear: the power increases proportionally with the concentration of Co^2+^ ions, having low values for these concentrations. However, for concentrations higher than 0.1 we delimited the range for the magnetic field to reasonable values in SPMHT, respectively up to 50 kA/m, because it is practically difficult to obtain large magnetic fields at frequencies of the order of 10^5^ Hz, and the magnetization of magnetic nanoparticles still remains in the superparamagnetic range.

In conclusion, taking into account all the above results and observations, in Table 7 we summarize the recommended values for the amplitude and frequency of the magnetic field depending on the concentration of Co^2+^ ions (x) in order to obtain SPMHT in optimal conditions, for maximum efficiency of the method. Thus, as a general observation it can be said that for lower x concentrations (0–0.1) the SPMHT is more suitable to be obtained for higher frequencies and lower magnetic fields, and for x concentrations higher than 0.1 the SPMHT becomes more suitable to be obtained at lower frequencies and higher magnetic fields.

Thus, under these conditions that we found the superparamagnetic hyperthermia with CoxFe_3−x_O_4_ nanoparticles can be applied with maximum efficiency in the destruction of tumor cells. In addition, if necessary in order to further increase the effectiveness of cancer therapy, superparamagnetic hyperthermia can be used in combination with other methods [5,12,16,17,43].

## 4. Conclusions

Using Co_x_Fe_3−x_O_4_ nanoparticles in SPMHT, by modifying the concentration x of Co^2+^ ions in the range x = 0–1 an efficient tuning can be obtained in a very wide range of values of observables/parameters of interest in SPMHT: maximum specific loss power (implicitly the heating temperature), the optimal diameter (size) of the nanoparticles corresponding to the maximum of specific loss power, the amplitude and the frequency of magnetic field. Thus, the optimal conditions for the application of SPMHT in the admissible biological limit can be found, depending on the concrete practical situations (tumor therapy in vitro, in vivo or in future clinical trials), to increase its efficiency in destroying tumor cells without affecting healthy tissues. Thus, the results obtained regarding the maximum specific loss power for the allowable biological limit (*P_sM_*)*_l_*, the optimal diameter of the nanoparticles for obtaining the maximum loss power (*D_Mo_*), the optimal range for the amplitude of alternating magnetic field (*H_o_*) and the limit frequencies (*f_l_*) (Table 6, Figure 8, Table 7) are effective data/tools for working in the practical implementation of SPMHT in order to obtain an increased efficacy in alternative therapy of tumors with as few toxicity as possible or even without toxicity.

## Figures and Tables

**Table 1:** Values of magnetocrystalline anisotropy constant, the spontaneous magnetisation, and the density at room temperature in the case of Co_x_Fe_3−x_O_4_ ferrite for different values of x in the range of 0–1.1 extracted from fit curves.

	Observables	x	*K* (×10^3^ J/m^3^)	*M_s_* (kA/m)	**ρ** (×10^3^ kg/m^3^)
No.					
1		0	11 ^(I)^	480 ^(I)^	5.24
2		0.05	38	477	~5.243
3		0.1	82	474	5.245
4		0.2	156	469	5.25
5		0.4	245	458	5.26
6		0.67	294	443	~5.27
7		0.8	290 ^(I)^	436	5.28
8		1	200 ^(II)^	425 ^(II)^	5.29
9		1.1	180 ^(I)^	-	-

^(I)^ [18]; ^(II)^ [19].

**Table 2:** Characteristic observables for Co_x_Fe_3−x_O_4_ nanoparticles and parameters of magnetic field.

Observables	*D* (nm)	*ε*	*H* (kA/m)	*f* (kHz)
Value range	1–20	0.1	10–50	100–500

**Table 3:** Maximum specific loss powers and corresponding diameter of nanoparticles as a function of the Co^2+^ ions concentration (x) for f = 100 kHz and H = 20 kA/m.

	Observables	x	*P_sM_* (W/g)	*D_M_* (nm)
No.				
1		0	31.69	17.4
2		0.05	21.14	11.5
3		0.1	11.85	9
4		0.2	6.45	7.2
5		0.4	3.96	6.2
6		0.67	3.05	5.9
7		0.8	3.03	5.9
8		1	4.11	6.7

**Table 4:** Maximum specific loss powers and corresponding diameter of nanoparticles as a function of the Co^2+^ ions concentration (x) for f = 250 kHz and H = 20 kA/m.

	Observables	x	*P_sM_* (W/g)	*D_M_* (nm)
No.				
1		0	77.6	16.7
2		0.05	48.95	11.1
3		0.1	26.59	8.6
4		0.2	14.23	6.9
5		0.4	8.66	6.
6		0.67	6.81	5.6
7		0.8	6.62	5.6
8		1	9.13	6.4

**Table 5:** Maximum specific loss powers and corresponding diameter of nanoparticles as a function of the Co^2+^ ions concentration (x) for f = 500 kHz and H = 20 kA/m.

	Observables	x	*P_sM_* (W/g)	*D_M_* (nm)
No.				
1		0	152.1	16.1
2		0.05	91.06	10.7
3		0.1	48.13	8.3
4		0.2	25.61	6.7
5		0.4	15.48	5.8
6		0.67	12.2	5.4
7		0.8	11.88	5.4
8		1	16.25	6.2

**Table 6:** Maximum specific loss power (*P_sM_*)*_l_* and corresponding diameter of nanoparticles (*D_Mo_*) in the case of Co_x_Fe_3−x_O_4_ ferrite nanoparticles for admissible biological limits *H* × *f* and different amplitudes of magnetic field *(H_o_*).

*H_o_* (kA/m)	*f_l_* (kHz)	*H* × *f* AHz/m	x = 0		x = 0.05		x = 0.1		x = 0.2		x = 0.4		x = 0.67		x = 0.8		x = 1	
			*(P_sM_)_l_* (W/g)	*D_Mo_* (nm)	*(P_sM_)_l_* (W/g)	*D_Mo_* (nm)	*(P_sM_)_l_* (W/g)	*D_Mo_* (nm)	*(P_sM_)_l_* (W/g)	*D_Mo_* (nm)	*(P_sM_)_l_* (W/g)	*D_Mo_* (nm)	*(P_sM_)_l_* (W/g)	*D_Mo_* (nm)	*(P_sM_)_l_* (W/g)	*D_Mo_* (nm)	*(P_sM_)_l_* (W/g)	*D_Mo_* (nm)
10	500	5 × 10^9^	62.29	16.1	26.13	10.7	12.47	8.3	6.47	6.7	3.89	5.8	3.06	5.4	2.98	5.4	4.08	6.2
20	250	5 × 10^9^	77.60	16.7	48.94	11.1	26.59	8.6	14.23	6.9	8.66	6.0	6.81	5.6	6.63	5.6	9.12	6.4
30	167	5 × 10^9^	82.57	17.0	62.52	11.3	39.12	8.7	20.07	7.1	13.75	6.1	10.77	5.7	10.44	5.7	14.41	6.5
40	125	5 × 10^9^	84.60	17.2	68.96	11.5	48.92	8.8	29.43	7.2	18.63	6.2	14.82	5.8	14.48	5.8	19.58	6.6
50	100	5 × 10^9^	85.94	17.4	74.31	11.5	56.68	8.9	36.59	7.2	23.78	6.2	18.52	5.9	18.42	5.9	24.31	6.7

**Table 7:** Optimum values of the amplitude and frequency of magnetic field for different concentration of Co^2+^ ions.

	Observables	x	*H_o_* (kA/m)	*f_l_* (kHz)
No.				
1		0	10–30	500–167
2		0.05	10–40	500–125
3		0.1	10–50	500–100
4		0.2	20–50	250–100
5		0.4; 1	30–50	167–100
6		0.67; 0.8	40–50	125–100

## Data Availability

Not applicable.
